# McHDV VP60 Virus-like Particles Elicit Protective Immunity Against *Moschus chrysogaster* Hemorrhagic Disease in Rabbits

**DOI:** 10.3390/pathogens13110925

**Published:** 2024-10-24

**Authors:** Yu Shao, Yudong Liu, Dong Huang, Qing Wang, Xiaoxiao He, Wenjing Zhao, Yunhai Zhao, Haiyun Ma, Xiaoyong Xing, Zhixiong Zhang, Shijun Bao

**Affiliations:** College of Veterinary Medicine, Gansu Agricultural University, Lanzhou 730070, China

**Keywords:** *Moschus chrysogaster* hemorrhagic disease virus (McHDV), virus like particles (VLPs), Codon bias, recombinant baculovirus, immunogenicity

## Abstract

*Moschus chrysogaster* viral hemorrhagic disease (McVHD), caused by the *Moschus chrysogaster* hemorrhagic disease virus (McHDV), is an acute and highly fatal infectious disease of musk deer. At present, there is no prevention or treatment for this disease. In this study, we constructed a recombinant bacmid containing the *McHDV VP60* gene and obtained the recombinant baculovirus rBac-McHDV VP60 by transfection into Sf9 (*Spodoptera frugiperda*) insect cells. The McHDV VP60 protein was successfully expressed in the insect cell-baculovirus expression system; furthermore, it was released in the supernatant of infected insect cells and spontaneously assembled to form virus-like particles (VLPs), which were structurally and immunologically indistinguishable from the *Moschus chrysogaster* viral hemorrhagic disease virion. Hypodermic vaccination of rabbits with the VLPs conferred complete protection in 14 days; this protection was found to be effective from the seventh day after VLP injection and was accompanied by a strong humoral response. This study is the first attempt to express the *VP60* gene of McHDV using an insect baculovirus system, which provides an experimental basis for the virus-like particle vaccine of McVHD.

## 1. Introduction

The *Moschus chrysogaster*, a member of the Musk family, is primarily distributed in India, Bhutan, Nepal, and China. In China, its distribution is mainly confined to the plateau regions of several northwestern provinces including Gansu, Qinghai, Sichuan, Ningxia, and Tibet [1]. This species has experienced a global population decline leading to its classification as endangered by the International Union for Conservation. Threats contributing to the potential extinction of this species include habitat destruction through deforestation and animal husbandry practices as well as biological exploitation for traditional medicine purposes [2,3]. To safeguard the survival prospects of these animals, China has established several national reserves. One such reserve is Xinglong Mountain National Nature Reserve located in Gansu Province. China’s first artificial breeding base for musk deer was established there in the early 1990s [4,5]. However, with the expansion of farming scale and changes in the lifestyle of this animal, diseases began to invade captive populations and gradually became an important factor restricting the further development of the species.

**Figure 1:**
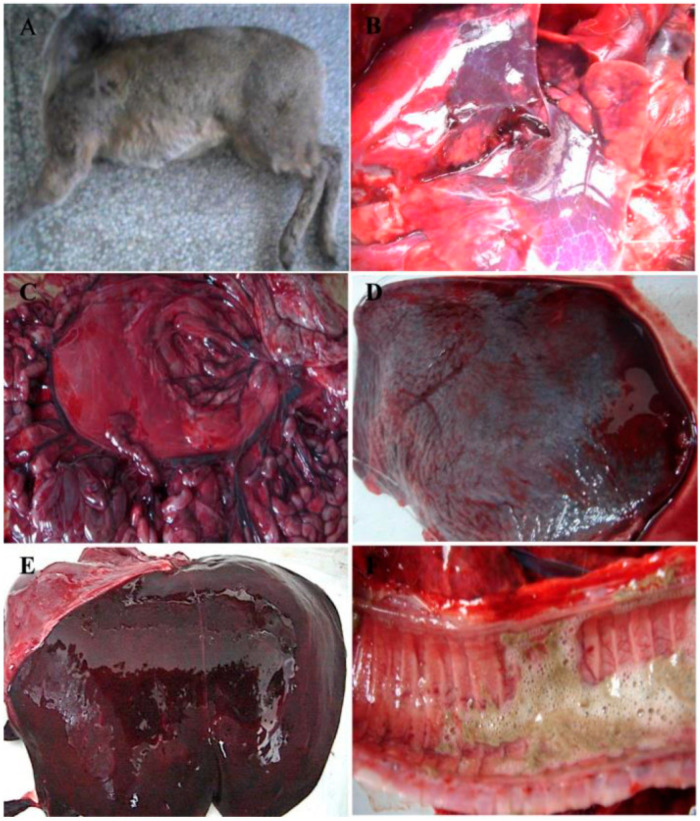
Macroscopic lesions observed in diseased Alpine musk deer. (**A**) A dead Alpine musk deer; (**B**) common signs of hyperemia, hemorrhage, and necrosis observed in the lung; (**C**) stomach and intestine; (**D**) spleen; (**E**) liver; (**F**) trachea [7].

**Figure 2:**
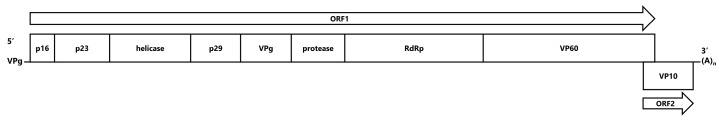
Structure of the virus genome.

*Moschus chrysogaster* viral hemorrhagic disease (McVHD), caused by the *Moschus chrysogaster* hemorrhagic disease virus (McHDV), is an acute and highly fatal infectious disease affecting musk deer. At the end of 2010 and the beginning of 2011, an outbreak occurred at a breeding farm within the Xinglong Mountain National Nature Reserve. Most of the affected deer developed an acute disease and suddenly died without visible clinical signs. The disease mainly occurred in younger animals within one year of age, with a case-fatality rate of approximately 100%. The animals with elongated disease processes showed symptoms of depression, anorexia, and opisthotonos. Postmortem examination of dead Alpine musk deer showed that hyperemia, hemorrhage, and necrosis were commonly present in organs including lung, liver, and spleen (Figure 1). To identify the potential pathogens, the Sequence-independent, single-primer amplification (SISPA) method was employed to amplify the gene fragments of the unknown pathogens, Sequencing analysis revealed that all obtained gene fragments were similar to RHDV genome fragments, with no detection of other viruses or bacteria. Additionally, transmission electron microscopy (TEM) was performed on negative staining samples prepared from tissue homogenates collected from deceased Alpine musk deer. Finally, the research team led by the author successfully isolated a calicivirus from deceased musk visceral tissue samples for the first time; with virus particles measuring approximately 35 nm in diameter without any envelope structure present. The complete genome sequence measured 7437 bp, which shared between 89.2–98.7% sequence identity with the Rabbit Hemorrhagic Disease Virus (RHDV) found on GenBank [6,7]. McHDV is a single-stranded positive-sense RNA virus, and its genome sequence is highly homologous to that of RHDV; therefore, it is classified as a member of the Lagovirus within the calicivirus family [8]. Referring to the gene structure of RHDV, the McHDV genome has two open reading frames (ORFs) named ORF1 and ORF2: ORF1 encodes a polyprotein, which is cleaved by the virus-encoded protease into several non-structural proteins and one structural protein (VP60), specifically arranged as p16, p23, helicase, p29, VPg, 3C-like protein, RNA-dependent RNA polymerase (RdRp), and structural protein VP60, while ORF2 encodes the capsid protein VP10 (Figure 2) [9].

In the face of such a significant threat, there is no effective treatment or prevention available for this disease currently. Therefore, it is imperative to urgently develop effective preventive vaccines for its control and prevention. Due to the inability to grow certain caliciviruses in cell culture, McHDV also lacks a cell culture system that allows viral replication. Ma et al. developed a tissue-inactivated vaccine, which demonstrated protection rates ranging from 66.7% to 100% in rabbits [10]. Bao et al. expressed the VP60 major epitopes of musk deer hemorrhagic disease virus in E.coli as tandem proteins, resulting in 100% immunity against the virulent virus when rabbits were immunized with purified VP60 protein [11]. Although the immunogenicity of VP60 against McVHD was confirmed, one limitation was that the VP60 protein was expressed as inclusion bodies.

In this study, we constructed a recombinant bacmid containing the *McHDV GS*/*YZ VP60* gene and successfully obtained the recombinant baculovirus rBac-McHDV VP60 by transfecting Sf9 insect cells. Subsequently, we expressed the capsid protein (VP60) of McHDV GS/YZ to generate virus-like particles (VLPs) and evaluated their immunogenicity in rabbits.

## 2. Materials and Methods

### 2.1. Plasmid, Cells, and Virus

The pFastBac1 plasmid was purchased from Beijing Qingke Biotechnology Co., Ltd., Beijing, China. Sf9 (*Spodoptera frugiperda*) cells were generously provided by Dr. Yi Ru from Lanzhou Veterinary Research Institute, Chinese Academy of Agricultural Sciences. The cells were cultured in an SIM SF Expression Medium (Sino Biological, Inc., Beijing, China, Cat.No.: MSF1) at a temperature of 27 °C without the addition of CO_2_. *E. coli* DH5α competent cells were purchased from Beijing TransGen Biotechnology Co., Ltd., Beijing, China. *E. coli* DH10Bac competent cells were purchased from Shanghai Angyu Biotechnology Co., Ltd., Shanghai, China. The virulent strain McHDV GS/YZ used in this study was isolated from diseased Alpine musk deer (*Moschus sifanicus*) in the Xinglong Mountain National Nature Reserve, Gansu Province, China. The genomic information of the GS/YZ strain has been published in GenBank (Acc. No. MN478485).

### 2.2. Construction of Recombinant Baculovirus

**Figure 3:**
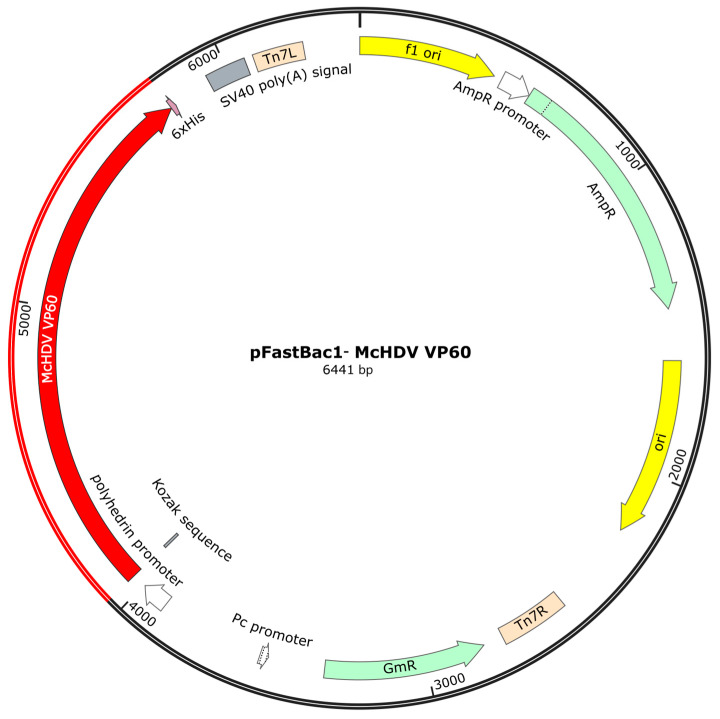
Schematic representation of the pFastBac1-*McHDV VP60* plasmid.

The nucleotide sequence of the *VP60* gene of the McHDV strain was optimized according to the codon bias of insect cells. In addition, the Kozak sequence and 6× His tag was added to the N terminus and C terminus, respectively. The above designed nucleotide sequence was synthesized and constructed into a pUC57 vector, named pUC57-*McHDV VP60*. The pUC57-*McHDV VP60* and pFastBac1 were digested with EcoR I and Hind III, and the target fragment was extracted by gel. The transfer plasmid pFastBac1-*McHDV VP60* was constructed, and the plasmid map is shown in Figure 3. The pFastBac1-*McHDV VP60* plasmid was transformed into Escherichia coli DH10Bac competent cells. After three rounds of blue and white spot-screening, the correct recombinant bacmid was identified by PCR and sequencing, which was named rBacmid-*McHDV VP60*. rBacmid-*McHDV VP60* was extracted using the Baculovirus Shuttle Vector Bacmid Mini Preparation Kit (Beyotime Biotech. Inc., Nantong, China, Cat.No.: D0031) and was mixed with Cellfection^®^ II Reagent (Gibco, New York, NY, USA, Cat.No.: 10362100) according to Bac-to-Bac Baculovirus Expression; then they were co-transfected into Sf9 insect cells and cultured at 27 °C for 3 to 5 days. After cytopathological changes were observed, the culture cells and supernatant were harvested and centrifuged at 1000× *g* for 20 min. The supernatant was the primary virus (P1 generation), named rBac-McHDV VP60.

### 2.3. Expression of McHDV VP60 Protein Was Identified by SDS-PAGE

Sf9 cells in the exponential growth phase were prepared at a density of 2 × 10^6^ cells/mL, and the P3 generation recombinant baculovirus was infected at MOI = 1. The cells were cultured at 27 °C for 3 to 5 days by suspension cultivation at 130 rpm, and 50 mL of the culture supernatant and cells were harvested. Then, 10mL was divided by centrifugation (1000× *g* for 10 min), and cells were resuspended in the same volume of phosphate buffer saline (PBS) as the culture supernatant and then lysed by sonication, followed by centrifugation to separate the cell supernatant and precipitate (15,000× *g* for 20 min), and the uninfected Sf9 cells and supernatant were used as negative controls. A total of 60 μL of the above samples and a 20 μL 4× loading buffer mixture were boiled in water for 10 min and then 20 μL of the sample was electrophoresed on a 10% polyacrylamide gel. SDS-PAGE was used to identify the protein expression level and to detect protein distribution.

### 2.4. Expression of McHDV VP60 Protein Was Identified by Western Blotting

The protein samples were subjected to 10% SDS-PAGE and transferred to the PVDF membrane. After blocking, incubating with the rabbit anti McHDV antibody, washing the membrane, incubating with the HRP-labeled goat anti-rabbit antibody, and washing the membrane, the recombinant protein was placed into a protein gel imager for exposure and color development with ECL detection reagent (Oriscience Biotechnology Co., Ltd., Chengdu, China, Cat. No.: PD202). The expressed recombinant protein was identified by Western blotting.

### 2.5. Transmission Electron Microscopy (TEM) Observation McHDV VLPs

Sf9 cells in the exponential growth phase were prepared at a density of 2 × 10^6^ cells/mL, and P3 generation recombinant baculovirus was infected at MOI = 1. The cells were cultured at 27 °C for 3 to 5 days at 130 rpm, and then the culture supernatant was collected after centrifugation (1000× *g* for 10min). 50mL culture supernatant was placed on ice, stirred continuously with a magnetic stirrater, and an equal volume of saturated ammonium sulfate solution was slowly added. After the ammonium sulfate was completely added, the solution was stirred for 4 h, and the precipitate was centrifuged at 15,000× *g* for 20 min. The supernatant was discarded, and the precipitate was resuspended with 5 mL of 0.05 M PBS. Then, 10 μL of the above protein solution was added to a 200-mesh gilder grid and adsorbed at room temperature for 2–3 min. The grid was blotted by filter paper, stained with 3% phosphotungstate acid, and observed by transmission electron microscope (JEM-1200EX, JEOL (Beijing) Co., Ltd., Beijing, China).

### 2.6. Animal Experiments

#### 2.6.1. Animals

For the trial, 10-week-old rabbits of both sexes from a commercial rabbit farm were used. All animals were clinically examined and the absence of antibodies against McHDV was verified by hemagglutination inhibition test(HI) [12]. The rabbits were fed with commercial rabbit food and water ad libitum.

#### 2.6.2. Vaccine Preparation

Sf9 cells were infected with the recombinant baculoviruses at MOI = 1 and incubated at 27 °C for 3–5 days. Then the cell cultures were harvested, and the blood coagulation (HA) test method for the determination of hemagglutination titer was used. The cell cultures were inactivated completely by adding diethylenimine (BEI) then diluted to a hemagglutination titer (HAU) of 10log2, 8log2, and 6log2 with 0.01 M PBS, respectively, and mixed with a sterilized aluminum hydroxide gel adjuvant at a ratio of 9:1.

#### 2.6.3. Immunization

Rabbits were randomly divided into four groups with four rabbits (2 rabbits of each sex) in each group. The rabbits in each group were immunized respectively via a subcutaneous injection with 0.5 mL vaccine prepared by the above method per rabbit or 0.5 mL PBS. Three groups of four rabbits were vaccinated with a single dose of 6log2 HAU, 8log2 HAU, or 10log2 HAU of vaccine, respectively. The PBS group was used as a blank control. After immunization, the health status of all rabbits was monitored repeatedly per day over two weeks. Blood serum samples were taken weekly and tested by hemagglutination Inhibition Test (HI). The animal experiment details are mentioned in Table 1.

#### 2.6.4. Challenge Infection

Rabbits were randomly divided into four groups with four rabbits (2 rabbits of each sex) in each group. And the immunization is as shown in Section 2.6.3. The challenge infection in all trials was performed by injection into subcutaneous tissue with 5log2 hemagglutination units (HAU) of the challenge McHDV virus GS/YZ strain. After the challenge, the health status of all rabbits was monitored repeatedly per day. All rabbits were challenged 14 days post-vaccination. At 10 days after the challenge, all remaining animals were euthanized in accordance with animal welfare regulations, and organ samples were taken and prepared for further analysis.

## 3. Results

### 3.1. Expression and Distribution of McHDV VP60 Protein Expression in Sf9 Cells

**Figure 4:**
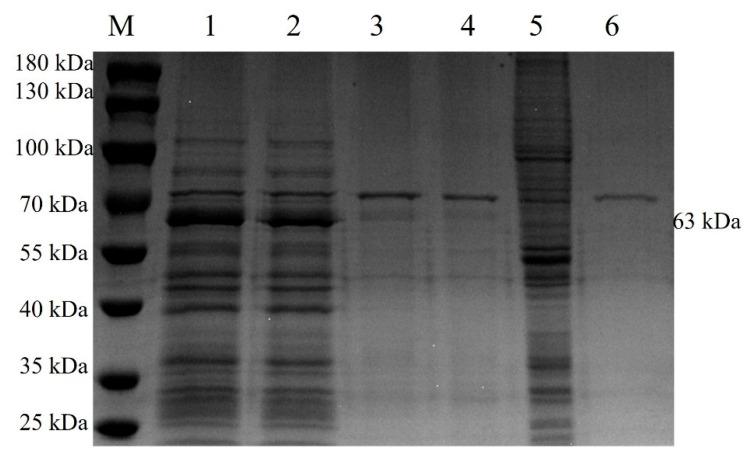
SDS-PAGE Analysis of VP60 Protein Expression from the Baculovirus Expression System. Culture supernatant and infected Sf9 cells were separated by centrifugation, and the cells obtained after centrifugation were resuspended in an equal volume of PBS as the culture supernatant and then subsequently lysed by sonication. After sonication, the resulting supernatant and precipitate were separated again by centrifugation. Lane M, protein marker; lane 1, the mixture of infected cells and culture supernatant; lane 2, culture supernatant after centrifugation; lane 3, supernatant from cells lysed by sonication; lane 4, precipitation from cells lysed by sonication; lane 5, non-infected sf9 cells; lane 6, culture medium control.

**Figure 5:**
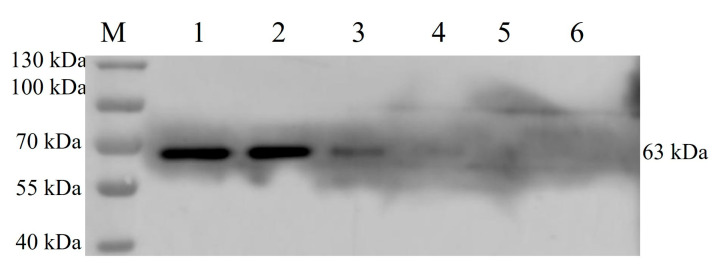
Western Blotting Analysis of VP60 Protein Expression from the Baculovirus Expression System. Lane M, protein marker; lane 1, the mixture of infected cells and culture supernatant; lane 2, culture supernatant after centrifugation; lane 3, supernatant from cells lysed by sonication; lane 4, precipitation from cells lysed by sonication; lane 5, non-infected sf9 cells; lane 6, culture medium control.

The cell suspension was collected after 5 days of rBac-McHDV VP60 culture, and then the supernatant and cells were collected separately by centrifugation. Cells were resuspended in the same volume of PBS as the culture supernatant and then lysed by sonication, followed by centrifugation to separate the cell supernatant and the precipitate. SDS-PAGE results showed that the target protein was present both in the culture supernatant and in the cells, and the culture supernatant contained the highest amount of target protein (Figure 4). Western blotting results showed a specific band at 63 kDa (Figure 5) with the expected size, indicating that the McHDV VP60 protein was correctly expressed in Sf9 cells.

### 3.2. Characterization of McHDV VLPs by Electron Microscopy

**Figure 6:**
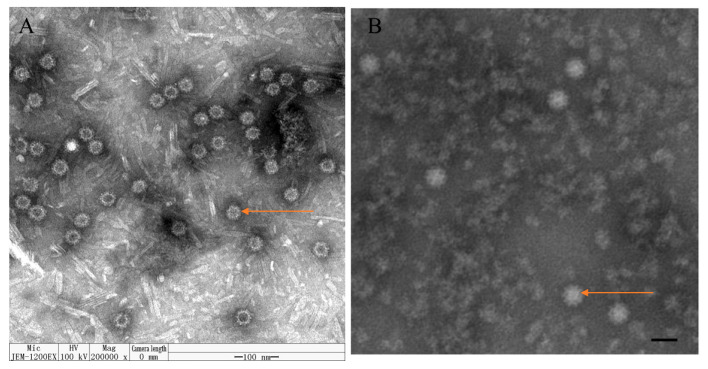
Electron micrographs of McHDV VLPs. (**A**) condensed McHDV VLPs (orange arrow). Bar: 100 nm. (**B**) native McHDV virions (orange arrow). Bar: 50 nm [7].

To determine whether the expressed McHDV VP60 protein self-assembled into VLPs, ammonium sulfate precipitation concentrated protein was visualized by transmission electron microscopy (TEM) analysis. As observed by TEM, the VP60 protein self-assembled into VLP with a size of about 35 nm and a spherical structure (Figure 6A), which was consistent with the morphological characteristics of native McHDV (Figure 6B). This demonstrates the successful assembly of McHDV VLPs in Sf9 insect cells.

### 3.3. Antigen Content Test of Vaccine

**Figure 7:**
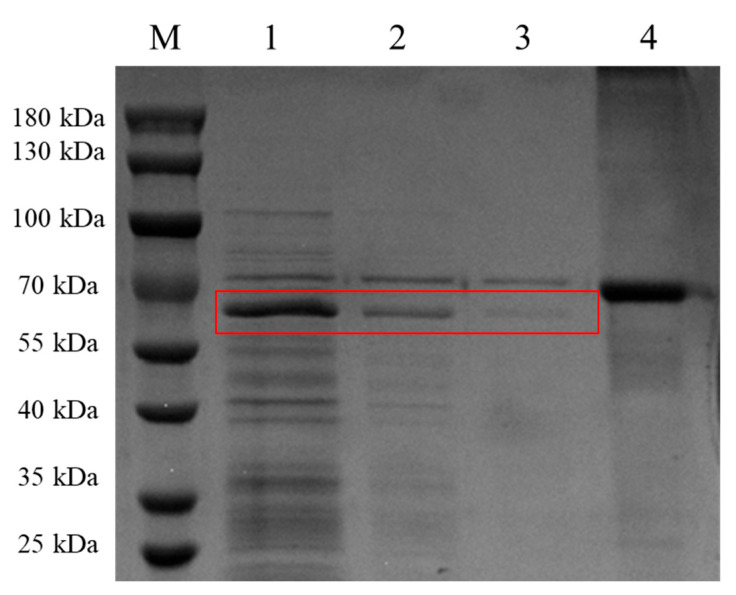
SDS-PAGE analysis of antigen content. Lane M, protein marker; lane 1, antigen at HAU of 10log2; lane 2, antigen at HAU of 8log2; lane 3, antigen at HAU of 6log2; lane 4, BSA at 1mg/mL. Note: Target proteins are shown in red boxes.

Sf9 cells were infected with the recombinant baculoviruses at MOI = 1 and incubated at 27 °C for 5 days, then the cell cultures were harvested. The Hemagglutination test showed that the hemagglutination titer (HAU) of virus-like particles was 12log2, and subsequently diluted to 10log2, 8log2, and 6log2 in a solution of 0.01M PBS. Confirmation of the target protein was achieved through SDS-PAGE analysis. Bovine serum albumin (BSA) at 1mg/mL was used as a reference, and the SDS-PAGE result showed that the antigen at HAU of 10log2, 8log2, and 6log2 decreased in a 2-fold gradient (Figure 7).

### 3.4. Immunogenicity of McHDV VLPs in Rabbits

**Figure 8:**
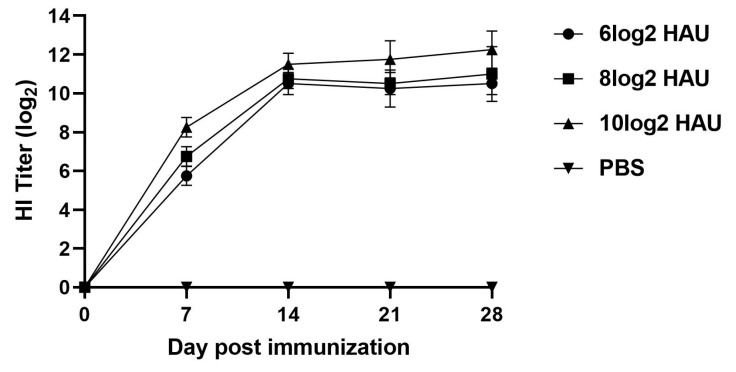
Serum antibody responses in rabbits after immunization with McHDV VLPs. Rabbit sera from different groups of immunized animals were collected at 0, 1, 2, 3, and 4 weeks post-immunization and then analyzed by HI.

The McHDV VLPs were then used to immunize rabbits. Groups of 4 rabbits were immunized subcutaneously with different dosages of VLPs or with PBS. Serum was collected every week and McHDV-specific antibodies in immunized rabbits were detected by the Hemagglutination Inhibition Test. The results showed that all rabbits were in good condition after immunization with normal food intake, and at the first week, HI antibody titers of rabbits immunized with 6log2 HAU, 8log2 HAU, and 10log2 HAU VLP groups were 6log2, 7log2, and 8log2, and it were shown to increase with time and were statistically different from the control group. Unexpectedly, the 3 different dose groups’ antibody levels did not differ much, and at 14 days, the HI antibody levels were at 9log2–11log2, and the antibody titers in rabbits immunized with 10log2 HAU VLPs were a little higher than those immunized with 8log2 HAU and 6log2 HAU VLPs (Figure 8).

### 3.5. McHDV VLPs Vaccine Can Protect Rabbits Against McHDV

The McHDV strain was used to challenge the immunized group and the control group 14 days after immunization. The results showed that all the rabbits of immunized groups survived, and all the rabbits of the control group died within 48 h. From the clinical manifestation, the control group all died with mouth or nose bleeding or without visible clinical signs, while the immune group showed normal clinical status, which suggested that the virus-like particle vaccine produced 100% immunity protection in rabbits (Table 2).

### 3.6. Macroscopic Examination of Organ Sampling after Challenge

**Figure 9:**
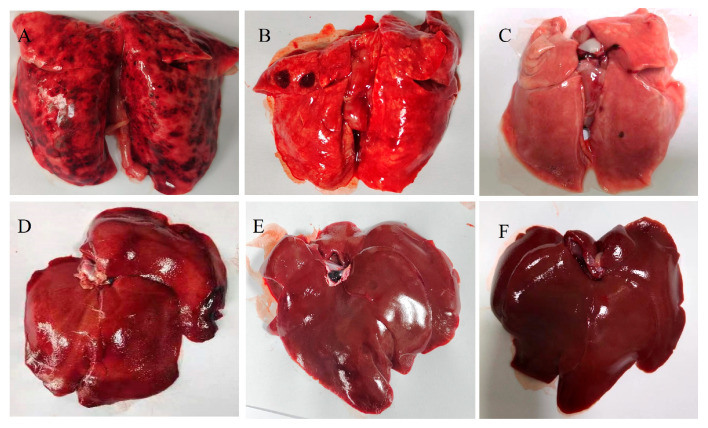
Macroscopic lesions observed in the rabbits’ lungs and livers. (**A**) Challenged death rabbit lung, (**B**) Live immune rabbit lung, (**C**) Healthy rabbit lung, (**D**) challenged death rabbit liver, (**E**) Live immune rabbit liver, (**F**) Healthy rabbit liver.

10 days after the challenge, all remaining animals were euthanized in accordance with animal welfare regulations, and organ samples were taken for macroscopic examination. The results showed that the organs of immunized rabbits were essentially identical to those of healthy rabbits (Figure 9C,F) in terms of size, color, and morphology, with no obvious pathological features observed (Figure 9B,E). In contrast, the negative control group exhibited diffuse black patches in the lungs (Figure 9A), and the livers appeared brittle with a dark-red color around the edges (Figure 9D).

### 3.7. Histopathology Examination of Organ Sampling after Challenge

**Figure 10:**
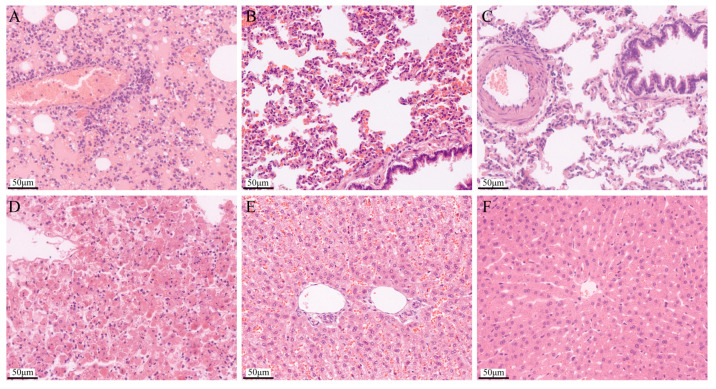
Macroscopic lesions observed in the rabbits’ lungs and livers. (**A**) Challenged death rabbit lung: Pulmonary telangiectasia and hemorrhage with serous exudation. (**B**) Live immune rabbit lung: Alveolar septal capillaries are congested with a small number of red blood cells in the alveolar space. (**C**) Healthy rabbit lung: The lungs were morphologically normal and structurally intact. (**D**) challenged death rabbit liver: The hepatocytes disintegrated, and their normal structure disappeared. (**E**) Live immune rabbit liver: The arrangement of the hepatic cords was disordered, and there were a large number of red blood cells in the space between the hepatic sinusoids. (**F**) Healthy rabbit liver: The hepatocytes were regularly arranged, and the structure and morphology were normal.

The histopathology examination results showed that compared with the normal lung tissue (Figure 10C), the lung tissue after challenge was the most seriously damaged, with pulmonary telangiectasia and bleeding, and serous exudation (Figure 10A). The immunized lung tissue showed slight damage, alveolar septal capillaries congested, and there were a few red blood cells in the alveolar space (Figure 10B). Compared with the normal liver tissue (Figure 10F), the liver cells in the challenged liver tissue disintegrated, the normal structure disappeared (Figure 10D), the liver cells in the immunized liver tissue were arranged disorderly, and there were a large number of red blood cells in the space of the liver sinusoid (Figure 10E).

## 4. Discussion

The *Moschus chrysogaster* viral hemorrhagic disease (McVHD) is an acute and highly fatal infectious disease of musk deer, which can result in significant economic losses. Therefore, it is imperative to control and prevent the spread of this disease. Our previous study revealed that McVHD is a calicivirus with a high degree of homology to the G2 RHDV gene sequence. However, it remains unknown whether the virus was transmitted across species due to mutations in the RHDV2 gene carried by hares. RHDV is believed to have very strict host specificity, and there is no evidence that the virus can infect animals other than rabbits either naturally or experimentally, nor is it known to cause disease in animals other than rabbits [13,14,15,16]. Therefore, to distinguish species specificity, we named the pathogen *Moschus chrysogaster* hemorrhagic disease virus (McHDV), and the disease caused by this pathogen was named *Moschus chrysogaster* viral hemorrhagic disease (McVHD) [6].Currently, there are no effective treatments or preventive measures available for this disease; hence, urgent efforts should be made towards developing effective preventive vaccines for its control.

Virus-like particles (VLPs) are hollow particles devoid of viral nucleic acid that are recombinantly expressed using one or more structural proteins from viruses in heterologous systems and self-assembly. They possess similar morphology to real virus particles but lack viral nucleic acid replication capability or infectivity, making them extremely safe as vaccine candidates [17]. Additionally, VLPs retain the spatial conformation of natural virus particles along with antigenic epitopes capable of inducing neutralizing antibodies. They can effectively interact with immune cells through mechanisms similar to those employed during actual viral infections and thereby elicit both humoral and cellular immune responses [18,19,20,21]. Due to their high safety profile and immunogenicity characteristics, VLPs represent a promising new genetic engineering subunit vaccine platform.

Studies have demonstrated that the expression of recombinant RHDV VP60 protein in insect cells leads to the formation of morphologically and antigenically identical VLPs resembling infectious RHDV virions [22,23,24,25]. These RHDV VLPs have been shown to confer complete protection against lethal challenges with RHDV in rabbits while also providing long-lasting immunity [26,27,28].

The results of our previous experiments on the pathogenicity of McHDV in rabbits have shown that all infected rabbits died within 72 h post-infection and exhibited clinical signs, including a sudden decrease in appetite, depression of activity, opisthotonos, and occlusion of the nares with foam and bloody secretions. Necropsy examination revealed congestion of the tracheal mucosa, pulmonary hyperemia, and carnification, as well as a friable, fatty, and discolored liver; congestion and enlargement of the spleen; and necrotic foci on the kidney [7]. These findings are similar to those observed in naturally infected musk deer. Therefore, rabbits can be used as model animals for studying vaccine immunogenicity.

In this study, a recombinant bacmid containing the *McHDV VP60* gene was constructed and transfected into Sf9 insect cells to obtain the recombinant baculovirus rBac-McHDV VP60. The expression of McHDV VP60 protein was successfully achieved in the insect cell-baculovirus expression system. Interestingly, McHDV VP60, which is a unique component of the McHDV capsid, was released in the supernatant of infected insect cells and self-assembled into virus-like particles (VLPs) without requiring any other viral components. These VLPs were structurally and immunologically indistinguishable from *Moschus chrysogaster* viral hemorrhagic disease virions. 

In our animal experiments, the rabbits were immunized with three different antigen doses (6log2, 8log2, or 10log2 HAU) to find the lowest protective dose of the vaccine. Unexpectedly, the lowest antigen dose group also provided complete protection to the rabbits, indicating that our vaccine had a good protective effect. In addition, the antibodies monitoring results show that at the first week, HI antibody titers of rabbits immunized with 6log2 HAU, 8log2 HAU, and 10log2 HAU VLP groups were 6log2, 7log2, and 8log2, and at the14th day, the HI antibody levels were all increased to 9log2–11log2, which also explains why all three dose groups survived the 14-day challenge protection test. 

The results of the present study demonstrated that the virus-like particle vaccine we developed effectively protected rabbits against lethal viral challenge. However, due to the national level I protection status of musk deer and the associated complex and costly approval process for immune evaluation, further assessment on this original animal was not conducted. Nonetheless, the nucleic acid sequence utilized in the virus-like particles is derived from musk deer, ensuring antigenic homology and providing a theoretical foundation for its efficacy on musk deer. Furthermore, considering the indispensability of preventing and controlling infectious diseases in musk deer with the industrialization of artificial breeding, future opportunities will be sought to complete immunogenicity studies on musk deer.

Laurent, S. et al. intramuscularly immunized rabbits with purified RHDV VLPs at a dose of 100 μg and showed complete protection 15 days after immunization [22]; The recombinant RHDV VP60 virus constructed by Marín, M.S. et al. [29] did not form virus-like particles; they obtained cell precipitates by centrifugation, lysed with lysates, and assessed for effective antigen concentration on SDS-PAGE. Results showed that the protective effect was dose dependent, as a dose of 10–25 μg provides 50% protection, and more than 50 μg doses achieve 100% protection [29]. Müller, C. et al. [26] centrifuged cell cultures with 1500× *g* at 4 °C for 20 min. resuspended in PBS, and ultrasound disintegrated the cells to prepare the vaccine [26]. These studies collectively demonstrate that cell extracts from uninfected Sf9 cells did not exhibit adverse effects on immunized animals; furthermore, they utilized clarified recombinant baculovirus-infected Sf9 cell lysate as the antigen rather than purified VP60.

In this study, through SDS-PAGE testing, we observed the presence of the target protein not only in cells but also in significant quantities in the medium. Considering the absence of serum in the medium, we directly collected and sonicated both cells and their medium before mixing them with aluminum adjuvant to prepare the vaccine. This approach offers several advantages: it simplifies mass production steps, eliminates the need for centrifugation during production, saves equipment and time, and further reduces costs. Furthermore, due to impurities present in our non-purified protein collection containing insect cell contaminants, quantifying immune dosage accurately based on protein concentration alone was unreliable. To effectively address this issue, we utilized the hemagglutination properties of virus-like particles to establish the effective dose as a benchmark for immunodosimetry. Simultaneous determination of antigen contents at corresponding hemagglutination titers by SDS-PAGE demonstrated that complete protection could be achieved through subcutaneous inoculation after 14 days with a dose of 6 log2HAU (equivalent to an effective antigen content of approximately 62.5 μg per rabbit) combined with an aluminum adjuvant. The findings from this experiment suggest that the hemagglutination titer test can serve as a reliable standard for determining immunization dosage, replacing SDS-PAGE.

Compared to the previous preparation process of virus-like particle vaccines, our innovative preparation process for obtaining virus-like particle vaccines is simple and convenient, eliminating the need for centrifugation and purification. Only the culture supernatant and cell mixture are collected by ultrasonication, with the immune dose determined based on the hemagglutination titer. On the one hand, our innovative process significantly improves antigen collection rates and reduces costs. On the other hand, it eliminates the requirement for large equipment and complex purification steps, making it more suitable for industrial production. 

Furthermore, compared to prokaryotic expression systems, our antigen preparation method effectively addresses various challenges encountered by prokaryotic cells during the expression of soluble proteins, such as endotoxin contamination and complexities in downstream purification.

## 5. Conclusions

In conclusion, we successfully constructed the recombinant baculovirus rBac-McHDV VP60 and demonstrated successful expression of the VP60 protein within insect cells using a baculovirus expression system, and the expressed VP60 can self-assemble into VLPs within these cells. Immunization with vaccines prepared using simple antigen production steps has been shown to confer complete protection in rabbits.

## Figures and Tables

**Table 1:** The immunization experimental designs used for the animal studies.

Groups	Number	Antigen	Dosage	Method	Time-Point for Sera Collection
1	4	6log2 HAU VLPs vaccine	0.5 mL	Subcutaneous	0 days before immunization; 1, 2, 3 and 4 weeks after immunization
2	4	8log2 HAU VLPs vaccine	0.5 mL	Subcutaneous	
3	4	10log2 HAU VLPs vaccine	0.5 mL	Subcutaneous	
4	4	PBS	0.5 mL	Subcutaneous	

**Table 2:** Details of protection experiment by challenge with virulent McHDV.

Groups	Number	Challenge Experiment		
		Death	Clinical Signs Rate *	Protection Rate
1	4	0	0%	4/4 (100%)
2	4	0	0%	4/4 (100%)
3	4	0	0%	4/4 (100%)
4	4	4	100%	0/4 (0%)

*: Clinical signs include a fall to the ground, limbs swimming, the breathing is forced, the body jerks back and forth, loud screaming, white or light-red mucus from the nostrils, and even death.

## Data Availability

The raw data supporting the conclusions of this article will be made available by the authors on request.
